# Serum zinc and dietary intake of zinc in relation to risk of different breast cancer subgroups and serum levels as a marker of intake: a prospective nested case-control study

**DOI:** 10.1007/s10549-021-06318-0

**Published:** 2021-07-05

**Authors:** Ylva Bengtsson, Malte Sandsveden, Signe Borgquist, Jonas Manjer

**Affiliations:** 1 Department of Surgery, Skåne University Hospital Malmö, Lund University, 20501 Malmö, Sweden; 2 Department of Oncology, Aarhus University Hospital, Aarhus University, Aarhus, Denmark; 3 Department of Clinical Sciences Lund, Division of Oncology, Division of Oncology and Pathology, Lund University and Skåne University Hospital, Lund, Sweden

**Keywords:** Breast cancer, Zinc, Subgroups, Serum, Diet

## Abstract

**Purpose:**

Zinc has been suggested to be protective against breast cancer, but the evidence remains inconclusive. One reason for inconsistent findings in previous studies may be that zinc only influences the risk of developing certain subtypes of breast cancer. Our study is the first study assessing zinc levels in relation to the risk of different breast cancer subgroups, defined by their tumor characteristics. In addition, we analyze serum zinc as a marker of dietary intake.

**Methods:**

The Malmö Diet and Cancer Study is a population-based cohort study that took place 1991–1996 in Malmö, Sweden. Until end of follow-up, 31 December 2013, 1186 incident cases were identified and matched to an equal number of controls. Odds ratios (ORs) for breast cancer, and having a certain tumor characteristic, were estimated in quartiles of baseline serum zinc and zinc intake and adjusted for potential confounders.

**Results:**

No associations were found between zinc, measured in serum or diet pre-diagnostically, and breast cancer risk. The adjusted OR for breast cancer in serum zinc Q4 compared to Q1 was 1.09 (0.85–1.41) and in zinc intake Q4 versus Q1 was 0.97 (0.77–1.23). Moreover, there were no clear associations between zinc and any breast cancer characteristics. The kappa value, 0.025 (*P* = 0.022), showed poor agreement between serum zinc and zinc intake.

**Conclusion:**

Our findings indicate that there is no clear association between zinc and overall breast cancer risk or risk of different breast cancer subgroups. Finally, our results suggest that serum zinc is a poor marker of zinc intake.

**Supplementary Information:**

The online version contains supplementary material available at 10.1007/s10549-021-06318-0.

## Introduction

Zinc has been suggested to play a role in breast cancer etiology, although previous studies regarding risk of breast cancer and zinc in serum and diet are inconclusive [1–5].

Zinc is an essential trace element, cofactor for more than 300 enzymes and needed for the growth and maintenance of the human body [6]. It is involved in numerous physiological processes such as RNA and DNA synthesis [7], cell proliferation and differentiation [8], redox regulation [9] and apoptosis [10]. Due to the effect of zinc in all these important processes in the cell, it has been hypothesized that it plays a role in defending against the development and progression of malignancy, although the mechanism of this role is not fully understood [11].

A meta-analysis by Jouybari et al. [1] concluded that there is a possible inverse association between zinc levels analyzed in plasma or serum and risk of breast cancer [1]. In contrast, another meta-analysis by Wu et al. [2] showed no difference in serum zinc level in breast cancer patients compared to healthy subjects [2]. The associations between zinc in diet and breast cancer risk has also been studied without conclusive results [3–5].

Breast cancer is a heterogenous disease, and one reason for inconsistent findings in previous studies may be that zinc only influences the risk of developing certain subtypes of breast cancer. It has been shown that zinc distribution and the zinc transporting network show unique profiles in breast cancer subgroups [12]. For instance, over-expression of zinc transporter ZIP6 has been noted in estrogen receptor positive (ER+) subtypes and is related to less aggressive tumors [13], and ZIP10 have been shown to be involved in invasive behavior of breast cancer cells [14].

To our knowledge, no epidemiological study has so far been made on serum or dietary intake of zinc in relation to risk of different breast cancer subgroups with different tumor characteristic. In addition, prior studies include few breast cancer cases and measure serum and dietary intake of zinc following diagnosis, not facing the issues of temporality which can arise when a disease process affects diet, metabolism and biomarkers. Further, no studies have been made using both serum and dietary intake of zinc as indicators of zinc status, which assess a broader dimension of the underlying nutrient of interest.

The present study is based on women form The Malmö diet and cancer study (MDCS), a population-based cohort study in Malmö with 17,035 women recruited between 1991 and 1996. Dietary assessment was collected with an interview-based diet history method and serum levels were analyzed from samples collected at baseline [15]. The current study is based on a follow-up identifying 1186 patients with incident breast cancer, and information on tumor characteristics was available for about 95% of these cases.

The aim of this study was to examine risk of breast tumors with different biological characteristics related to pre-diagnostic levels of serum zinc and dietary intake of zinc using a nested case-control design within The MDCS. An additional aim was to study serum zinc as a marker of dietary intake of zinc.

## Material and methods

### The Malmö diet and cancer study (MDCS)

The study population consists of women enrolled in the MDCS, which is a prospective population-based cohort study in Malmö, Sweden. The MDCS and the baseline investigations have been described in detail elsewhere [15, 16]. Briefly, the baseline examination took place between 1991 and 1996, and include a dietary assessment, blood samples and a self-administered questionnaire. The questionnaire included questions regarding socioeconomic status, medical history, lifestyle habits and for women also menopausal status and reproductive history. Height, weight, body composition and blood pressure were assessed by physical examination.

Women born between 1923 and 1950 were invited to participate. Consequently, this resulted in a total female cohort of 17,035 women, representing a participation rate of 43% for women.

### Identification of breast cancer cases and controls

By record linkage with the Swedish Cancer Registry, breast cancer cases until December 31st, 2013 were identified. Women diagnosed with breast cancer prior to inclusion in the study were excluded (*n* = 576), resulting in a total of 1186 eligible incident cases. Using two different selection methods, an identical number of controls (*n* = 1186) were included in the study. Approximately half of the controls were chosen based on a previous study by Almquist et al. [17] where incidence density matching was used to match each case to a subject at risk at the time of case occurrence. Using age as a time scale, controls were matched on menopausal status, time of inclusion and age. Among the matched controls, 694 remained breast cancer free until December 31st, 2013. To make it an equal number of cases and controls, the remaining controls (*n* = 492) were selected from a randomized subsample of the MDCS, the cardiovascular cohort [18]. The CV-subcohort includes 3531 women that completed the baseline examination. Of these, 2615 women remained breast cancer free up to 31 December 2013. Subsequently, 492 women were selected randomly, making it a total of 1186 controls. The purpose of choosing the cardiovascular cohort was that genetic material was available as needed in a parallel future study.

**Fig. 1:**
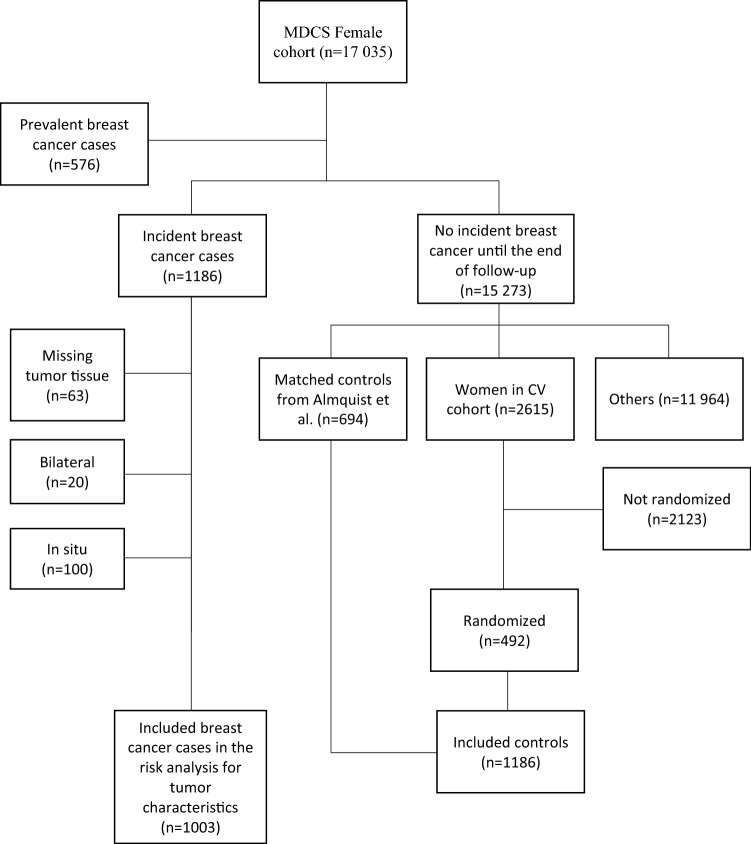
Flow chart of inclusion and exclusion of the cases and controls. Adapted from Sandsveden et al. [19]

As a consequence of missing tumor material, 63 patients were excluded from risk analyses of tumor characteristics. In addition, twenty cases had bilateral breast cancer and were excluded due to problems in deciding which side to be used in analyses of histopathology, receptor status, lymph node status and tumor size. Further, 100 cases were excluded from the risk analyses of tumor characteristics as a result of tumor data showing carcinoma in situ. A flowchart, adapted from Sandsveden et al. [19, 20], of patient inclusion and exclusion in the study is illustrated in Fig. 1.

### Blood sample collection and laboratory analysis

Venipuncture was done on non-fasting participants at baseline. Serum was extracted within one hour of blood sample collection and afterwards stored at − 80 °C. In October 2015, the saved serum zinc was analyzed by ALS Scandinavia AB, Luleå, Sweden, as previously described by Sandsveden et al. [21]. Serum samples were analyzed on ICP-SFMS (Thermo Element 2) using single-element standard solutions, NIST, traceable to the International System of Units. An amount of 0.15 mL was mixed with an alkaline liquid containing 0.1% ammonia and 0.005% EDTA/Triton-X to at quantity of 10 mL. Seronorm, obtained from Sero AS, Norway (Lot 0608414), was analyzed together with the serum samples as a reference material. The detection limit of zinc was 10 ng/mL, and the inter-assay coefficient of variation was 3.3%. Albumin had previously been analyzed as part of another study [17].

Due to insufficient amount of saved serum from 262 women, these women were reported as having missing serum zinc data and were consequently excluded from the analyses using serum zinc as an indicator of zinc status.

### Dietary assessment method

The methodology used in The MDCS have good ranking compared to a reference method consisting of 18 days of weighed food records [20]. As previously described more in detail [20, 22], it consists of three parts: (a) a 168-item semi quantitative diet history questionnaire gathering information about the overall meal pattern, e.g., potion-size and frequency of foods consumed regularly (b) a 7-day food diary for registration of cooked meals, beverages, nutrient supplements, pharmaceutical drugs and natural remedies (c) a 45–60 min diet history interview where portion sizes and cooking preparations in the questionnaire and menu book were described more in detail. Based on the estimate of portion sizes and frequencies from the questionnaire and the food diary, a mean daily intake of foods was determined. To translate the food intake to nutrient and energy intake the PCKost2-93 from the National Food Administration in Uppsala, Sweden, was used. PCKost2-93 contains roughly 1600 basic foods with additional food codes and recipes added specially for the MDCS. In the present study, the sum of food intake and supplemental intake of zinc are expressed as dietary intake of zinc.

### Histopathological analyses

Data on tumor characteristics in the identified breast tumors were collected in three separate time periods. (1) Tumor material from cases diagnosed until 31st December 2004, as described in two previous studies [23, 24], were re-evaluated by a senior breast pathologist concerning the histopathological diagnosis, i.e., histological type in agreement with the world health organization classification guidelines [25] and histological grade according to Elston and Ellis [26]. In addition, micro array (TMA) was constructed to re-evaluate proliferation (Ki67), human epidermal growth factor 2 (HER2)- and hormone receptor status. (2) Likewise, for cases diagnosed from 2005 to December 31, 2007, TMA was used to re-evaluate Ki67, HER2- and hormone receptor status [27]. However, histological grade and type were gathered from medical records and pathology reports. (3) From December 31, 2007, information about tumor characteristics was exclusively collected from medical records. During all periods, data on tumor size and nodal status were gathered from hospital records.

HER2 status was gathered either from immunohistochemical (IHC) score (1991–2004), or from regional and national cancer registries and hospital records which comprised analysis data from both in situ hybridization (ISH) and IHC (2005–2013). Breast tumors were considered HER2 positive when ISH was amplified or when scored as 3+ on the IHC staining [28]. HER2 was regarded as negative when ISH was not amplified or when scored as 0 or 1+ on the IHC staining. Breast tumors with a score of 2+ was classified as missing if no data from ISH amplification was available. Tumors were divided, based on the expression of Ki67, into tertiles (low, intermediate or high) separately for each time period 1991–2004, 2005–2007 and 2008–2013 [19]. ER- and progesterone receptor (PgR) status were evaluated according to the nucleus expression of ER and PgR and tumors were dichotomized into negative (≤ 10% staining intensity) and positive (≥ 10% staining intensity) [23].

### Breast cancer surrogate intrinsic subtypes classification

Based on ER, PR, and HER2 receptor status along with histologic grade and Ki67 positivity, surrogate intrinsic subtypes were created [19, 29]. Tumors were divided into *Luminal A-like* (ER+ and HER2- with (a) grade 1 or (b) grade 2 and low Ki67 or (c) grade 2, intermediate Ki67 and PgR+), *Luminal B-like* (ER+ and HER2- with (a) grade 3 or (b) grade 2 and high Ki67 or (c) grade 2 intermediate Ki67 and PgR-), *HER2-positive* (all tumors classified as HER2 positive), or *triple-negative (TNBC)* (all tumors that were ER-, PgR- and HER2-).

### Statistical method

Dietary intake of zinc was adjusted for total energy intake using the residual model. The residual is the difference between the actual zinc intake and the predicted zinc intake in a regression model with total energy intake as the independent variable and absolute zinc intake as the dependent variable.

Subsequently, the study population was divided into groups according to quartiles (Q) of serum zinc levels and dietary intake of zinc. Quartile cut-offs were based of the distribution of all women in our study, both cases and controls. Quartiles of residuals in our study are presented as the median of total dietary intake of zinc.

To measure the level of agreement between serum zinc and dietary intake of zinc, Cohen’s Kappa coefficient with a p-value was calculated from a cross table of serum zinc and dietary intake of zinc quartiles.

Logistic regression was used to estimate odds ratios (ORs) and 95% confidence intervals (CIs) for breast cancer and having a certain tumor characteristic in different quartiles of serum zinc and zinc intake. Thereafter, the same analyses were done for dichotomized groups of serum zinc and zinc intake, defined as low (a merge of Q1 and Q2) and high (a merge of Q3 and Q4), and for groups combining low and high serum zinc and dietary intake of zinc: low serum + low intake, high serum + low intake, low serum + high intake and high serum + high intake. Furthermore, trends over quartiles of serum zinc and zinc intake were calculated by introducing the quartile number as a continuous variable in the Cox’s proportional hazard model.

In a second model, all analyses were adjusted for factors with at least five percentage points difference between cases and controls in supplementary table S1: Age, socioeconomic index, use of oral contraceptives, menopausal status at baseline, use of hormone replacement therapy (HRT) and year sample was taken.

A case–case analysis was used to examine heterogeneity between different breast cancer subgroups regarding their association with serum zinc and zinc intake. An logistic regression analysis was applied, and p values of 0.05 or less were considered to be statistically significant.

Several sensitivity analyses were conducted. In the analyses using dietary intake of zinc as an indicator for zinc status, a sensitivity analysis was performed adjusting for the abovementioned factors plus season of collection of dietary data, interviewer who conducted the diet history interview and dietary method before and after September 1st, 1994, but not adjusting for baseline year. In the analyses using serum zinc, a sensitivity analysis was done adjusting for factors with at least five percentage points difference in supplementary table S1 plus time of year sample was taken. An additional sensitivity analysis was made excluding incident cases occurring within the two first years of follow-up. Since phosphorus have been shown to affect the availability of zinc absorption [30], all analyses were additionally adjusted for intake of phosphorus, both from diet and supplements. Phosphorus was adjusted for total energy intake using the residual method, as described above. Finally, because 70% of all serum zinc in bound to albumin [6], a subsample, consisting of 694 cases and 788 controls, was used to conduct a sensitivity analysis in the analyses with serum zinc, adjusting for factors with at least five percentage points difference in supplementary table S1 plus albumin levels.

All analyses were performed using SPSS Statistics version 25.

## Results

### Baseline characteristics

Cases were younger, more often premenopausal, more likely to have low socioeconomic index, to have used oral contraceptives and HRT and to have their blood samples taken in 1994–1996 compared to controls (Supplementary table S1).

Women with the highest serum zinc levels (Q4) were more likely to be older, postmenopausal, to have a lower educational degree, have a BMI ≥ 30 kg/m^2^ and to have their blood sample collected during winter compared to women with the lowest serum zinc levels (Q1) (Table 1). In addition, women in Q4 were less likely to have had used oral contraceptives and HRT compared to women in Q1 (Table 1).

**Table 1:** Established potential risk factors for breast cancer and serum zinc levels

	Serum zinc				Missing	Total
	1 (*N* = 528)	2 (*N* = 527)	3 (*N* = 530)	4 (*N* = 525)		
	≤ 599.0 ng/ml	599.1–673.5 ng/ml	673.6–751.0 ng/ml	≥ 751.1 ng/ml	(*N* = 262)	(*N* = 2372)
Age						
< 50	28.8	26.8	21.9	17.1	13.4	22.5
50–55	24.4	21.8	27.0	24.4	16.4	23.5
55–60	18.2	19.5	18.7	25.0	17.9	20.1
≥ 60	28.6	31.9	32.5	33.5	52.3	33.9
Socioeconomic index						
Manual	35.4	37.2	33.4	38.9	38.9	36.5
Non-manual	58.7	55.0	59.1	52.6	56.9	56.4
Employer	5.3	7.0	6.8	7.0	3.8	6.2
Missing	0.6	0.8	0.8	1.5	0.4	0.8
Education						
O-level college	66.5	66.8	65.7	71.4	79.4	68.9
A-level college	9.1	7.8	5.8	7.0	6.5	7.3
University	24.2	25.2	28.5	21.0	14.1	23.6
Married or cohabiting						
No	32.8	30.4	34.3	33.9	29.8	32.5
Yes	67.2	69.6	65.7	66.1	70.2	67.5
Parity						
1	20.1	18.4	20.6	19.2	22.5	19.9
2	43.8	45.0	41.3	44.0	40.1	43.1
3	16.7	16.1	15.7	15.0	17.2	16.0
4 or more	4.5	5.7	6.0	4.2	5.3	5.1
Nullipara	13.1	12.7	12.1	14.5	12.2	13.0
Missing	1.9	2.1	4.3	3.0	2.7	2.8
Age at first childbirth						
≤ 20	17.8	17.3	15.5	14.9	18.3	16.6
21–25	34.7	33.8	34.0	36.2	35.1	34.7
26–30	22.3	24.5	24.9	22.9	22.9	23.6
≥ 31	10.2	9.5	9.2	8.6	8.8	9.3
Age at menarche						
≤ 12	21.6	22.6	20.9	24.3	21.4	22.2
13–14	54.2	50.5	55.1	51.1	55.6	53.0
≥ 15	24.2	27.0	24.0	24.7	23.0	24.7
Ever use of oral contraceptives						
No	42.8	47.1	51.3	52.6	53.8	49.0
Yes	57.2	52.9	48.7	47.4	45.8	50.9
Menopausal status						
Pre	33.5	30.9	24.3	21.1	15.3	26.1
Peri	8.5	7.4	11.1	7.8	3.8	8.2
Post	58.0	61.7	64.5	71.0	80.9	65.7
HRT, current						
No	74.4	74.2	77.9	80.8	80.9	77.3
Yes	25.4	25.6	21.5	19.0	18.7	22.4
Alcohol consumption (g/d)						
0	4.2	6.3	8.1	7.6	8.8	6.8
< 15	63.8	62.0	65.5	63.8	64.5	63.9
15–30	17.6	14.8	12.3	13.3	10.7	14.1
> 30	3.6	3.4	2.6	3.2	3.4	3.2
Infrequent	10.8	13.5	11.5	11.8	12.6	12.0
BMI (kg/m^2^)						
< 20	5.1	5.5	4.9	5.1	3.8	5.0
20–25	50.0	48.6	50.9	43.8	42.7	47.7
25–30	34.8	34.2	32.8	35.4	34.7	34.4
≥ 30	10.0	11.8	11.3	15.6	18.7	12.9
Time of year sample was taken						
Spring	29.2	28.8	24.5	26.1	29.0	27.4
Summer	19.7	15.0	12.6	11.4	17.6	15.0
Autumn	31.4	31.1	34.3	32.0	37.0	32.8
Winter	19.7	25.0	28.5	30.5	16.4	24.9
Year sample was taken						
1991	7.8	7.4	10.0	11.0	12.6	9.4
1992	26.7	21.6	20.8	30.9	12.2	23.6
1993	20.6	24.1	17.9	25.7	26.7	24.8
1994	13.4	15.7	15.3	15.4	18.3	15.3
1995	17.8	19.5	17.0	10.9	19.5	16.7
1996	13.6	11.6	9.1	6.1	10.7	10.2

All data are presented as column percentage. Missing data < 1% is not shown

However, women with the highest dietary intake of zinc (Q4) were slightly younger, more likely to have a higher educational degree, to be younger at menarche, to have had used oral contraceptives and to have dietary data collected during January-March compared to women with the lowest dietary intake of zinc (Q1) (Supplementary table S2).

Women with missing serum zinc values were older and more often postmenopausal, less educated, less likely to have had used oral contraceptives and more often obese (Table 1).

### Serum zinc as a biomarker for dietary intake of zinc

Dietary intake of zinc and serum zinc were compared as presented in Table 2. The row percentage for increasing serum zinc quartiles in women with the highest dietary intake of zinc (Q4) was similar: 20.4, 22.1, 22.4 and 24.1. The kappa value, 0.025 (*P* = 0.022), showed poor agreement between serum zinc and dietary intake of zinc.

**Table 2:** Serum zinc and dietary intake of zinc

	Median^a^ (ug/day)	Serum zinc				Missing	Total
		1 (*n* = 528)	2 (*n* = 527)	3 (*n* = 530)	4 (*n* = 525)		
		≤ 599.0 ng/ml	599.1–673.5 ng/ml	673.6–751.0 ng/ml	≥ 751.1 ng/ml	(*n* = 262)	
Dietary intake of zinc							
1 (*n* = 592)	8.8	141 (23.8)	126 (21.3)	120 (20.3)	135 (22.8)	70 (11.8)	592 (100.0)
2 (*n* = 594)	9.2	119 (20.0)	147 (24.7)	135 (22.7)	128 (21.5)	65 (10.9)	594 (100.0)
3 (*n* = 593)	10.5	147 (24.8)	123 (20.7)	142 (23.9)	119 (20.1)	62 (10.5)	593 (100.0)
4 (*n* = 593)	19.9	121 (20.4)	131 (22.1)	133 (22.4)	143 (24.1)	65 (11.0)	593 (100.0)
Total (*n* = 2372)		528 (22.3)	527 (22.2)	530 (22.3)	525 (22.1)	262 (11.0)	2372 (100.0)

The data shown in brackets are presented as row percentage

^a^Residuals of zinc intake quartiles are presented as the median of total dietary intake of zinc

### Serum zinc, dietary intake of zinc and breast cancer risk

No overall association was seen between zinc and breast cancer risk. This was found both in the analyzes using serum zinc, dietary intake of zinc and a combination of serum and intake of zinc as an indicator of zinc status (Table 3). The adjusted OR for breast cancer in serum zinc Q4 compared to Q1 was 1.09 (0.85–1.41) (*P*_trend_ = 0.64) and adjusted OR for breast cancer in zinc intake Q4 versus Q1 was 0.97 (0.77–1.23) (*P*_trend_ = 0.64) (Table 3). Similarly, adjusted OR for breast cancer in the group with high serum and dietary intake of zinc compared to the group with low serum and dietary intake of zinc was 0.94 (0.73–1.21) (Supplementary table S3). Further adjustment for season, interviewer and dietary method in the multivariate analyses did not considerably alter the results (data not shown), and neither did excluding incident cases occurring within the first year of follow-up (*n* = 10) (data not shown). Likewise, when repeating the analyses with the additional adjustment for phosphor intake, similar results were seen both in the analyses using serum zinc and dietary intake of zinc as an indicator for zinc status; the adjusted OR:s for breast cancer in serum zinc and zinc intake Q4 versus Q1 were 1.09 (0.85–1.41) and 0.96 (0.73–1.26), respectively. Finally, the results were not significantly altered when analyzing a subsample including albumin as an additional factor in the multivariate analyses; adjusted OR for breast cancer in serum zinc Q4 versus Q1 was 1.05 (0.75–1.47).

**Table 3:** Odds ratio (OR) for cases and controls in relation to quartiles of serum zinc levels and dietary intake of zinc as compared to the first quartile and group

Quartile	Serum zinc				Dietary intake of zinc			
	Interval (ng/ml)	Case/controls	Crude OR (95 CI)	Adjusted^a^ OR (95 CI)	Median^b^ (ug/day)	Case/controls	Crude OR (95 CI)	Adjusted^a^ OR (95 CI)
1	≤ 599.0	269/259	1.00	1.00	8.8	298/294	1.00	1.00
2	599.1–673.5	275/252	1.05 (0.83–1.34)	1.06 (0.83–1.37)	9.2	307/287	1.05 (0.84–1.33)	1.06 (0.84–1.35)
3	673.6–751.0	259/271	0.92 (0.72–1.17)	1.00 (0.77–1.28)	10.5	292/301	0.96 (0.76–1.20)	0.97 (0.76–1.22)
4	≥ 751.1	253/272	0.90 (0.70–1.14)	1.09 (0.85–1.41)	19.9	289/304	0.94 (0.75–1.18)	0.97 (0.77–1.23)
P-trend			0.24	0.64			0.43	0.64
Group								
1	≤ 673.5	544/511	1.00	1.00	9.0	605/581	1.00	1.00
2	≥ 673.5	512/543	0.89 (0.75–1.05)	1.01 (0.84–1.21)	12.8	581/605	0.92 (0.79–1.08)	0.94 (0.79–1.11)

^a^Adjusted for age, socioeconomic index, use of oral contraceptives, hormone replacement therapy, menopausal status and year of inclusion

^b^Residuals of zinc intake quartiles are presented as the median of total dietary intake of zinc

### Serum zinc, dietary intake of zinc and breast cancer subgroups

As presented in Tables 4 and 5, there were no clear associations between serum zinc, dietary intake of zinc and risk for any specific tumor characteristic or intrinsic subtype. For ER-negative tumors the adjusted OR, in the analyses using dietary intake of zinc, for Q2 versus Q1 was 1.99 (1.08–3.67) (Table 5), for tumors with intermediate Ki67 adjusted OR for Q3 versus Q1 was 0.64 (0.43–0.97) (Table 5) and for TNBC adjusted OR for Q2 versus Q1 was 3.06 (1.40–6.71) (Table 5). The association was verified in the heterogeneity analysis for ER, Ki67 and TNBC (Table 5). For all other tumor characteristic or intrinsic subtypes no associations with breast cancer risk were found (Tables 4 and 5). Similarly, no associations were seen for the dichotomized groups with high serum and dietary intake of zinc compared to the groups with low serum and dietary intake of zinc (Supplementary table S4 and S5). Adjusted OR:s were similar in all the sensitivity analyses described above (data not shown).

**Table 4:** Odds ratios (OR) for breast cancer clinical features and quartiles of serum zinc levels and dietary intake of zinc as compared to the first quartile

Tumor characteristics	Serum zinc^a^			Dietary intake of zinc^a^		
	Case/controls	Crude OR (95 CI)	Adjusted^b^ OR (95 CI)	Case/controls	Crude OR (95 CI)	Adjusted^b^ OR (95 CI)
Lymph node positive^c^						
1	60/259	1.00	1.00	85/294	1.00	1.00
2	71/252	1.22 (0.83–1.79)	1.21 (0.81–1.81)	63/287	0.76 (0.53–1.09)^d^	0.71 (0.48–1.03)^d^
3	66/271	1.05 (0.71–1.55)	1.25 (0.83–1.87)	73/301	0.84 (0.59–1.19)	0.83 (0.57–1.19)
4	62/272	0.98 (0.66–1.46)	1.29 (0.85–1.95)	73/304	0.83 (0.58–1.18)	0.83 (0.58–1.20)
Lymph node negative						
1	137/259	1.00	1.00	140/294	1.00	1.00
2	140/252	1.05 (0.78–1.41)	1.08 (0.79–1.46)	177/287	1.30 (0.98–1.71)	1.32 (0.99–1.75)
3	135/271	0.94 (0.70–1.26)	1.00 (0.73–1.35)	153/301	1.07 (0.81–1.41)	1.06 (0.80–1.42)
4	139/272	0.97 (0.72–1.29)	1.16 (0.86–1.58)	147/304	1.02 (0.77–1.35)	1.03 (0.77–1.38)
Tumor size ≤ 20 mm^c^						
1	149/259	1.00	1.00	159/294	1.00	1.00
2	154/252	1.06 (0.80–1.41)	1.09 (0.81–1.46)	185/287	1.19 (0.91–1.56)	1.22 (0.92–1.61)
3	143/271	0.92 (0.69–1.22)	0.99 (0.73–1.34)	166/301	1.02 (0.78–1.34)	1.02 (0.77–1.35)
4	156/272	1.00 (0.75–1.32)	1.24 (0.92–1.67)	172/304	1.05 (0.80–1.37)	1.06 (0.80–1.41)
Tumor size > 20 mm						
1	58/259	1.00	1.00	82/294	1.00	1.00
2	70/252	1.24 (0.84–1.83)	1.21 (0.81–1.80)	70/287	0.87 (0.61–1.25)	0.83 (0.57–1.20)
3	70/271	1.15 (0.78–1.70)	1.24 (0.83–1.85)	69/301	0.82 (0.57–1.18)	0.81 (0.56–1.17)
4	56/272	0.92 (0.61–1.38)	1.10 (0.72–1.66)	66/304	0.78 (0.54–1.12)	0.78 (0.54–1.14)
Grade 1^c^						
1	60/259	1.00	1.00	64/294	1.00	1.00
2	62/252	1.06 (0.72–1.58)	1.05 (0.70–1.59)	64/287	1.02 (0.70–1.50)	1.02 (0.68–1.52)
3	61/271	0.97 (0.66–1.44)	1.04 (0.69–1.57)	54/301	0.82 (0.56–1.23)	0.85 (0.56–1.28)
4	41/272	0.65 (0.42–1.00)	0.81 (0.52–1.27)	77/304	1.16 (0.81–1.68)	1.17 (0.80–1.73)
Grade 2						
1	97/259	1.00	1.00	111/294	1.00	1.00
2	98/252	1.04 (0.75–1.44)	1.06 (0.75–1.49)	123/287	1.14 (0.84–1.54)	1.15 (0.84–1.58)
3	98/271	0.97 (0.70–1.34)	1.05 (0.75–1.48)	115/301	1.01 (0.75–1.38)	1.00 (0.73–1.37)
4	111/272	1.09 (0.79–1.50)^d^	1.32 (0.94–1.85)	106/304	0.92 (0.68–1.26)	0.93 (0.67–1.28)
Grade 3						
1	51/259	1.00	1.00	65/294	1.00	1.00
2	61/252	1.23 (0.82–1.85)	1.22 (0.80–1.87)	68/287	1.07 (0.74–1.56)	1.01 (0.68–1.49)
3	53/271	0.99 (0.65–1.51)	1.08 (0.70–1.67)	65/301	0.98 (0.67–1.43)	0.94 (0.64–1.40)
4	59/272	1.10 (0.73–1.66)	1.36 (0.88–2.09)^d^	54/304	0.80 (0.54–1.19)	0.80 (0.53–1.20)

^a^Serum zinc quartiles and quartiles of dietary intake of zinc as in Table 3

^b^Adjusted for age, socioeconomic index, use of oral contraceptives, hormone replacement therapy, menopausal status and year of inclusion

^c^Reference group in the heterogeneity analysis

^d^Statistically significant results in the heterogeneity analysis (*p* < 0.05)

**Table 5:** Odds ratios (OR) for breast cancer subgroups and quartiles of serum zinc levels and dietary intake of zinc as compared to the first quartile

	Serum zinc^a^			Dietary intake of zinc^a^		
Tumor characteristics*	Case/controls	Crude OR (95 CI)	Adjusted^b^ OR (95 CI)	Case/controls	Crude OR (95 CI)	Adjusted^b^ OR (95 CI)
ER+^c^						
1	176/259	1.00	1.00	211/294	1.00	1.00
2	193/252	1.13 (0.86–1.47)	1.15 (0.87–1.52)	199/287	0.97 (0.75–1.24)	0.99 (0.76–1.28)
3	183/271	0.99 (0.76–1.30)	1.08 (0.82–1.43)	200/301	0.93 (0.72–1.19)	0.95 (0.73–1.23)
4	171/272	0.93 (0.71–1.21)	1.16 (0.87–1.54)	205/304	0.94 (0.73–1.21)	0.98 (0.75–1.27)
ER-						
1	21/259	1.00	1.00	17/294	1.00	1.00
2	21/252	1.03 (0.55–1.93)	1.08 (0.57–2.04)	34/287	2.05 (1.12–3.75)^d^	1.99 (1.08–3.67)^d^
3	17/271	0.77 (0.40–1.50)	0.85 (0.43–1.67)	26/301	1.49 (0.79–2.81)	1.36 (0.72–2.59)
4	30/272	1.36 (0.76–2.44)	1.53 (0.84–2.79)	23/304	1.31 (0.69–2.50)	1.27 (0.66–2.45)
Low ki67^c^						
1	70/259	1.00	1.00	72/294	1.00	1.00
2	74/252	1.09 (0.75–1.57)	1.08 (0.73–1.60)	75/287	1.07 (0.74–1.53)	1.04 (0.71–1.52)
3	56/271	0.77 (0.52–1.13)	0.84 (0.56–1.27)	83/301	1.13 (0.79–1.61)	1.10 (0.76–1.60)
4	67/272	0.91 (0.63–1.33)	1.21 (0.82–1.80)	76/304	1.02 (0.71–1.46)	1.00 (0.69–1.45)
Intermediate Ki67						
1	52/259	1.00	1.00	71/294	1.00	1.00
2	52/252	1.03 (0.67–1.57)	1.05 (0.68–1.61)	58/287	0.84 (0.57–1.23)	0.84 (0.57–1.24)
3	58/271	1.07 (0.71–1.61)	1.07 (0.70–1.64)	47/301	0.65 (0.43–0.97)^d^	0.64 (0.43–0.97)^d^
4	51/272	0.93 (0.61–1.42)	1.02 (0.66–1.58)	69/304	0.94 (0.65–1.36)	0.96 (0.66–1.40)
High ki67						
1	46/259	1.00	1.00	46/294	1.00	1.00
2	49/252	1.10 (0.71–1.70)	1.08 (0.69–1.70)	66/287	1.47 (0.98–2.22)	1.42 (0.93–2.16)
3	49/271	1.02 (0.66–1.58)	1.14 (0.72–1.79)	60/301	1.27 (0.84–1.93)	1.23 (0.80–1.89)
4	56/272	1.16 (0.76–1.77)	1.40 (0.90–2.19)	55/304	1.16 (0.76–1.77)	1.17 (0.76–1.81)
Luminal A-like^c^						
1	90/259	1.00	1.00	113/294	1.00	1.00
2	103/252	1.18 (0.84–1.64)	1.20 (0.84–1.69)	102/287	0.93 (0.68–1.27)	0.95 (0.68–1.31)
3	96/271	1.02 (0.73–1.42)	1.11 (0.78–1.57)	101/301	0.87 (0.64–1.19)	0.89 (0.64–1.23)
4	84/272	0.89 (0.63–1.25)	1.17 (0.82–1.68)	111/304	0.95 (0.70–1.29)	0.97 (0.71–1.34)
Luminal B-like						
1	39/259	1.00	1.00	50/294	1.00	1.00
2	45/252	1.19 (0.75–1.88)	1.17 (0.73–1.88)	46/287	0.94 (0.61–1.45)	0.91 (0.59–1.42)
3	48/271	1.18 (0.75–1.86)	1.26 (0.79–2.02)	46/301	0.90 (0.58–1.38)	0.88 (0.57–1.38)
4	41/272	1.00 (0.63–1.60)	1.21 (0.74–1.97)	52/304	1.01 (0.66–1.53)	1.03 (0.67–1.59)
HER2+						
1	22/259	1.00	1.00	16/294	1.00	1.00
2	21/252	0.98 (0.53–1.83)	1.00 (0.53–1.89)	26/287	1.67 (0.88–3.17)	1.48 (0.77–2.85)
3	12/271	0.52 (0.25–1.08)	0.56 (0.27–1.16)	24/301	1.47 (0.76–2.81)	1.37 (0.71–2.67)
4	18/272	0.78 (0.41–1.49)	0.97 (0.50–1.89)	13/304	0.79 (0.37–1.66)	0.75 (0.35–1.61)
TNBC						
1	17/259	1.00	1.00	9/294	1.00	1.00
2	14/252	0.85 (0.41–1.75)	0.90 (0.43–1.88)	27/287	3.07 (1.42–6.65)^d^	3.06 (1.40–6.71)^d^
3	8/271	0.45 (0.19–1.06)	0.49 (0.21–1.18)	18/301	1.95 (0.86–4.42)	1.75 (0.76–4.01)
4	24/272	1.34 (0.71–2.56)	1.49 (0.77–2.90)	14/304	1.50 (0.64–3.53)	1.45 (0.61–3.44)

^a^Serum zinc quartiles and quartiles of dietary intake of zinc as in Table 3

^b^Adjusted for age, socioeconomic index, use of oral contraceptives, hormone replacement therapy, menopausal status and year of inclusion

^c^Reference group in the heterogeneity analysis

^d^Statistically significant results in the heterogeneity analysis (*p* < 0.05)

## Discussion

In this study, no associations were found between pre-diagnostic levels of serum zinc, or dietary intake of zinc, and breast cancer risk. In addition, no clear associations were found between the abovementioned factors and any specific breast cancer characteristics or intrinsic subtype. Finally, poor agreement was seen between serum and dietary intake of zinc.

The potential cancer preventive effects of zinc have been suggested to be mediated through a wide range of mechanisms including regulation of apoptosis [10] and of transcription factors involved in the progression of tumors [31]. Indeed, experimental studies suggest that low zinc intake can suppress *N*-methyl-*N*-nitrosourea–induced mammary tumorigenesis in rats [32]. However, previous research in humans is inconclusive [1–5].

Two recent meta-analyses on breast cancer risk in relation to serum zinc showed contradictory results [1, 2]. Although meta-analyses combine many studies, these two studies used relatively small sample sizes with 776 and 662 invasive breast cancer cases, respectively. In addition, zinc was measured following diagnosis and a strong evidence of heterogeneity in both studies was seen. Likewise, inconsistent results have been shown in two case-control studies using dietary intake of zinc as an indicator of zinc status. One case-control study from Germany including 310 cases showed an inverse association between dietary intake of zinc and breast cancer risk [4], while the other case-control study with 2362 cases from Canada presented no such relationship [5]. However, the Canadian study indicated that supplementation of zinc for 10 or more years is associated with reductions in breast cancer risk [5].

This is the first study assessing zinc levels in relation to the risk of different breast cancer subgroups, defined by their tumor characteristics. Previous studies have revealed a subgroup‐dependent pattern of zinc distribution and zinc transporter expression [12–14, 33]. For example, Chandler et al. observed homogenous distribution of zinc in TNBC tumors, whereas luminal tumors showed high zinc accumulation around the margins [12]. In addition, it has been shown by Farquharson et al. that zinc concentrations were higher in ER+ tumor tissues compared to ER- tumor tissues [33]. However, our results indicate that there are no clear associations between zinc in serum or diet and any breast cancer characteristics or intrinsic subtype.

As far as we know, this is the first study made measuring serum and dietary intake of zinc pre-diagnostically. It cannot be excluded that serum zinc levels measured following diagnosis are affected by cancer therapy or by the disease itself. Consequently, breast cancer did most probably not affect serum zinc levels in the current study. In addition, we performed a sensitivity analysis excluding incident cases occurring within the first two years of follow-up and found that this did not substantially alter odds ratios. Hence, it is not likely that manifest or subclinical disease affect the findings of our study.

Furthermore, we believe that the present study has several additional strengths. First, we have a long follow-up period (18–23 years) and a relatively large sample size which gives the study good statistical power. Second, we use a modified diet history method which have been validated [34] and provide a detailed exposure assessment of zinc-containing foods, beverages and supplements [20]. Third, the MDCS data set contained information on many potential confounding factors that were adjusted for.

However, a number of important limitations need to be considered. For instance, there are some well-known potential problems of subgroups analyses – false negatives due to low power and false positives due to multiple testing [35]. Compared to the power of the overall risk analyses, some of our subgroup analyses have much less statistical power, e.g., the TNBC group which only include 63 cases. In addition, the differences in odds of certain subgroups among women with low and high zink intake might be false positives due to chance alone. To illustrate, we found an association between zinc intake in Q2 versus Q1 and ER-negative tumors, zinc intake in Q2 versus Q1 and TNBC and an inverse association between zinc intake in Q3 versus Q1 and intermediate Ki67. It is possible that the results of ER- and TNBC subgroups are overlapping since ER- tumors are part of the TNBC tumors. Consequently, as a result of many subgroup analyses and the absence of a clear pattern in our results, we cannot rule out chance as the explanation. Another potential limitation is that the MDCS had an overall participation rate of 43% for women, which make selection bias a potential issue. However, the MDCS has similar sociodemographic characteristics and prevalence of smoking and obesity as participants in a mailed health survey in the same population with a participation rate of 74.6% [16]. Further limitations concern venipuncture, which was done on non-fasting participants, and the fact that time of day for blood donation was not recorded. This could potentially increase the within subject variation since serum zinc concentrations might fluctuate up to 20% during a 24 h period [36], mainly due to effects of food intake [37]. On the other hand, other factors potentially affecting serum zinc levels, such as phosphorus and albumin levels, were taken into consideration in the sensitivity analyses. In addition, the majority of women in our study have a serum zinc concentration within the normal reference range (0.66 to 1.10 µg/mL) [38].

Our results showed poor agreement between serum zinc and dietary intake of zinc. This finding are in agreement with a previously reported meta-analysis by Lowe et al. [39]. The meta-analysis, based on twenty-four estimates among 2469 participants, showed that doubling on zinc intake in adults increases serum/plasma concentrations by only 6% [39]. Taken together with our results, this supports the fact that effective homeostatic regulations exist to prevent deviations in serum zinc when dietary intake of zinc fluctuate [40, 41]. Furthermore, the plasma pool of zinc is relatively small and can be easily influenced by minor changes in tissue zinc [40]. In addition, many factors have been identified to have possible effects on serum zinc concentration unrelated to dietary intake of zinc, for example infection and inflammation [42], time of day [36], inhibitors of zinc absorption such as phosphorus in the form of phytate [30] and levels of albumin [38]. Altogether, the search continues for a reliable and simple indicator if zinc status. Still, plasma/serum zinc is currently the most widely used biomarker of zinc status [43], even though serum/plasma zinc appears to more useful indicator of zinc status under more extreme dietary conditions [41].

## Conclusion

No associations were found between zinc levels measured in serum or diet pre-diagnostically and breast cancer risk. In addition, there were no associations between zinc in serum or diet and any breast cancer characteristics or intrinsic subtype. Finally, poor agreement was seen between serum zinc and dietary intake of zinc.

## Supplementary Information

Below is the link to the electronic supplementary material.

Supplementary file1 (DOCX 17 kb)

Supplementary file2 (DOCX 20 kb)

Supplementary file3 (DOCX 25 kb)

Supplementary file4 (DOCX 23 kb)

Supplementary file5 (DOCX 14 kb)

## Data Availability

The data will be shared on reasonable request to the corresponding author.

## Abbreviations

BMI: Body mass index
CI: Confidence interval
ER: Estrogen receptor
HER2: Human epidermal growth factor receptor 2
HRT: Hormone replacement therapy
IHC: Immunohistochemistry
ISH: In situ hybridization
MDCS: The Malmö diet and cancer study
OR: Odds ratio
PgR: Progesterone receptor
Q: Quartile
TMA: Tissue microarray
TNBC: Triple negative breast cancer
ZIP: Zinc-regulated transporters, Iron-regulated transporter-like Proteins
