# Supplementary material for: Serum zinc and dietary intake of zinc in relation to risk of different breast cancer subgroups and serum levels as a marker of intake: a prospective nested case-control study
Source: Breast Cancer Res Treat. 2021 Jul 5;189(2):571–83. doi: 10.1007/s10549-021-06318-0 (PMC8357733; doi:10.1007/s10549-021-06318-0)
Supplement: Supplementary file 1 — Supplementary file1 (DOCX 17 kb) [file 10549_2021_6318_MOESM1_ESM.docx]

Supplementary table 4**.** Odds Ratios (OR) for breast cancer clinical features and two groups of serum zinc levels and dietary intake of zinc as compared to the first group

|  |  | Serum zinc^a^ | | |  | Dietary intake of zinc^a^ | | |
| --- | --- | --- | --- | --- | --- | --- | --- | --- |
| Tumor characteristics |  | Case/  controls | Crude OR (95 CI^b^) | Adjusted^c^ OR (95 CI) |  | Case/  controls | Crude OR (95 CI) | Adjusted^c^ OR (95 CI) |
| Lymph node positive |  |  |  |  |  |  |  |  |
| 1 |  | 131/511 | 1.00 | 1.00 |  | 148/581 | 1.00 | 1.00 |
| 2 |  | 128/543 | 0.92 (0.70-1.21) | 1.15 (0.86-1.53) |  | 146/605 | 0.95 (0.73-1.22) | 0.97 (0.75-1.27) |
| Lymph node negative |  |  |  |  |  |  |  |  |
| 1 |  | 277/511 | 1.00 | 1.00 |  | 317/581 | 1.00 | 1.00 |
| 2 |  | 274/543 | 0.93 (0.76-1.14) | 1.04 (0.84-1.29) |  | 300/605 | 0.91 (0.75-1.10) | 0.90 (0.74-1.11) |
| Tumor size ≤20 mm |  |  |  |  |  |  |  |  |
| 1 |  | 303/511 | 1.00 | 1.00 |  | 344/581 | 1.00 | 1.00 |
| 2 |  | 299/543 | 0.93 (0.76-1.14) | 1.06 (0.86-1.31) |  | 338/605 | 0.94 (0.78-1.14) | 0.94 (0.77-1.14) |
| Tumor size >20 mm |  |  |  |  |  |  |  |  |
| 1 |  | 128/511 | 1.00 | 1.00 |  | 152/581 | 1.00 | 1.00 |
| 2 |  | 126/543 | 0.93 (0.70-1.22) | 1.06 (0.80-1.41) |  | 135/605 | 0.85 (0.66-1.10) | 0.87 (0.67-1.14) |
| Grade 1 |  |  |  |  |  |  |  |  |
| 1 |  | 122/511 | 1.00 | 1.00 |  | 128/581 | 1.00 | 1.00 |
| 2 |  | 102/543 | 0.79 (0.59-1.05) | 0.91 (0.67-1.23) |  | 131/605 | 0.98 (0.75-1.29) | 1.00 (0.76-1.33) |
| Grade 2 |  |  |  |  |  |  |  |  |
| 1 |  | 195/511 | 1.00 | 1.00 |  | 234/581 | 1.00 | 1.00 |
| 2 |  | 209/543 | 1.01 (0.80-1.27) | 1.14 (0.90-1.45) |  | 221/605 | 0.91 (0.73-1.13) | 0.90 (0.72-1.12) |
| Grade 3 |  |  |  |  |  |  |  |  |
| 1 |  | 112/511 | 1.00 | 1.00 |  | 133/581 | 1.00 | 1.00 |
| 2 |  | 112/543 | 0.94 (0.71-1.26) | 1.09 (0.80-1.48) |  | 119/605 | 0.86 (0.65-1.13) | 0.87 (0.65-1.15) |

^a^Serum zinc quartiles and quartiles of dietary intake of zinc as in table 3

^b^Adjusted for age, socioeconomic index, use of oral contraceptives, hormone replacement therapy, menopausal status and year of inclusion
