# Supplementary material for: Serum zinc and dietary intake of zinc in relation to risk of different breast cancer subgroups and serum levels as a marker of intake: a prospective nested case-control study
Source: Breast Cancer Res Treat. 2021 Jul 5;189(2):571–83. doi: 10.1007/s10549-021-06318-0 (PMC8357733; doi:10.1007/s10549-021-06318-0)
Supplement: Supplementary file 2 — Supplementary file2 (DOCX 20 kb) [file 10549_2021_6318_MOESM2_ESM.docx]

Supplementary table S5**.** Odds Ratios (OR) for breast cancer subgroups and two groups of serum zinc levels and dietary intake of zinc as compared to the first group

|  |  | Serum zinc^a^ | | |  | Dietary intake of zinc^a^ | | |
| --- | --- | --- | --- | --- | --- | --- | --- | --- |
| Tumor characteristics |  | Case/  controls | Crude OR (95 CI) | Adjusted^b^ OR (95 CI) |  | Case/  controls | Crude OR (95 CI) | Adjusted^b^ OR (95 CI) |
| ER+ |  | 369/511 | 1.00 | 1.00 |  | 410/581 | 1.00 | 1.00 |
| 1 |  | 354/543 | 0.90 (0.75-1.09) | 1.04 (0.85-1.27) |  | 405/605 | 0.95 (0.79-1.13) | 0.97 (0.81-1.17) |
| 2 |  |  |  |  |  |  |  |  |
| ER- |  | 42/511 | 1.00 | 1.00 |  | 51/581 | 1.00 | 1.00 |
| 1 |  | 47/543 | 1.05 (0.68-1.62) | 1.14 (0.73-1.78) |  | 49/605 | 0.92 (0.61-1.39) | 0.88 (0.58-1.33) |
| 2 |  | 369/511 | 1.00 | 1.00 |  | 410/581 | 1.00 | 1.00 |
| Low Ki67 |  |  |  |  |  |  |  |  |
| 1 |  | 144/511 | 1.00 | 1.00 |  | 147/581 | 1.00 | 1.00 |
| 2 |  | 123/543 | 0.80 (0.61-1.05) | 0.97 (0.73-1.29) |  | 159/605 | 1.04 (0.81-1.34) | 1.03 (0.79-1.34) |
| Intermediate Ki67 |  |  |  |  |  |  |  |  |
| 1 |  | 104/511 | 1.00 | 1.00 |  | 129/581 | 1.00 | 1.00 |
| 2 |  | 109/543 | 0.99 (0.74-1.32) | 1.03 (0.76-1.39) |  | 116/605 | 0.86 (0.66-1.14) | 0.87 (0.65-1.15) |
| High Ki67 |  |  |  |  |  |  |  |  |
| 1 |  | 95/511 | 1.00 | 1.00 |  | 112/581 | 1.00 | 1.00 |
| 2 |  | 105/543 | 1.04 (0.77-1.41) | 1.21 (0.88-1.67) |  | 115/605 | 0.99 (0.74-1.31) | 0.99 (0.74-1.33) |
| Luminal A-like |  |  |  |  |  |  |  |  |
| 1 |  | 193/511 | 1.00 | 1.00 |  | 215/581 | 1.00 | 1.00 |
| 2 |  | 180/543 | 0.88 (0.69-1.11) | 1.04 (0.81-1.33) |  | 212/605 | 0.95 (0.76-1.18) | 0.96 (0.76-1.21) |
| Luminal B-like |  |  |  |  |  |  |  |  |
| 1 |  | 84/511 | 1.00 | 1.00 |  | 96/581 | 1.00 | 1.00 |
| 2 |  | 89/543 | 1.00 (0.72-1.38) | 1.14 (0.82-1.60) |  | 98/605 | 0.98 (0.72-1.33) | 1.00 (0.73-1.37) |
| HER2+ |  |  |  |  |  |  |  |  |
| 1 |  | 43/511 | 1.00 | 1.00 |  | 42/581 | 1.00 | 1.00 |
| 2 |  | 30/543 | 0.66 (0.41-1.06) | 0.75 (0.46-1.22) |  | 37/605 | 0.85 (0.54-1.34) | 0.85 (0.54-1.36) |
| TNBC |  |  |  |  |  |  |  |  |
| 1 |  | 31/511 | 1.00 | 1.00 |  | 36/581 | 1.00 | 1.00 |
| 2 |  | 32/543 | 0.97 (0.58-1.62) | 1.03 (0.61-1.73) |  | 32/605 | 0.85 (0.52-1.39) | 0.79 (0.48-1.30) |

^a^Serum zinc quartiles and quartiles of dietary intake of zinc as in table 3

^b^Adjusted for age, socioeconomic index, use of oral contraceptives, hormone replacement therapy, menopausal status and year of inclusion
