# Supplementary material for: Serum zinc and dietary intake of zinc in relation to risk of different breast cancer subgroups and serum levels as a marker of intake: a prospective nested case-control study
Source: Breast Cancer Res Treat. 2021 Jul 5;189(2):571–83. doi: 10.1007/s10549-021-06318-0 (PMC8357733; doi:10.1007/s10549-021-06318-0)
Supplement: Supplementary file 3 — Supplementary file3 (DOCX 25 kb) [file 10549_2021_6318_MOESM3_ESM.docx]

Supplementary table S1. Percentage distribution cases, controls and excluded groups

|  | | Controls  (n=1186) | Invasive tumors with tumor material  (n=1003) | Carcinoma in situ  (n=100) | Bilateral cancers  (n=20) | Invasive tumors with missing material  (n=63) | Total  (n=2372) |
| --- | --- | --- | --- | --- | --- | --- | --- |
| Age | <50 | 19.2 | 25.8 | 27.0 | 15.0 | 27.0 | 22.5 |
|  | 50-55 | 23.5 | 23.4 | 34.0 | 5.0 | 14.3 | 23.5 |
|  | 55-60 | 20.9 | 19.5 | 11.0 | 25.0 | 25.4 | 20.1 |
|  | ≥60 | 36.3 | 31.2 | 28.0 | 55.0 | 33.3 | 33.9 |
| Socio-economic index | Manual | 40.1 | 33.6 | 24.0 | 45.0 | 31.7 | 36.5 |
|  | Non-manual | 52.4 | 59.6 | 67.0 | 55.0 | 63.5 | 56.4 |
|  | Employer | 6.8 | 5.9 | 6.0 | 0.0 | 3.2 | 6.2 |
|  | Missing | 0.6 | 0.9 | 3.0 | 0.0 | 1.6 | 0.8 |
| Education | O-level college | 71.2 | 68.0 | 55.0 | 70.0 | 61.9 | 68.9 |
|  | A-level college | 7.8 | 6.9 | 6.0 | 5.0 | 9.5 | 7.3 |
|  | University | 20.9 | 24.8 | 39.0 | 25.0 | 28.6 | 23.6 |
| Married or cohabiting | No | 32.1 | 33.4 | 26.0 | 15.0 | 33.4 | 32.5 |
|  | Yes | 67.9 | 66.6 | 74.0 | 85.0 | 66.6 | 67.5 |
| Parity | 1 | 20.8 | 19.0 | 18.0 | 19.2 | 19.0 | 19.9 |
|  | 2 | 41.0 | 45.1 | 46.0 | 44.0 | 47.6 | 43.1 |
|  | 3 | 16.9 | 15.7 | 14.0 | 15.0 | 9.5 | 16.0 |
|  | 4 or more | 5.6 | 4.5 | 3.0 | 4.2 | 4.8 | 5.1 |
|  | Nullipara | 12.0 | 13.9 | 16.0 | 14.5 | 15.9 | 13.0 |
|  | Missing | 3.6 | 1.9 | 3.0 | 3.0 | 3.2 | 2.8 |
| Age at first childbirth | ≤20 | 17.5 | 16.0 | 10.0 | 20.0 | 19.0 | 16.6 |
|  | 21-25 | 34.7 | 35.0 | 35.0 | 30.0 | 31.7 | 34.7 |
|  | 26-30 | 23.4 | 23.5 | 26.0 | 35.0 | 19.0 | 23.6 |
|  | ≥31 | 8.9 | 9.7 | 10.0 | 10.0 | 11.1 | 9.3 |
|  | Missing | 3.6 | 2.0 | 3.0 | 0.0 | 3.2 | 2.9 |
| Age at menarche | ≤12 | 20.7 | 23.6 | 29.3 | 5.0 | 23.0 | 22.2 |
|  | 13-14 | 53.0 | 51.8 | 55.6 | 70.0 | 63.9 | 53.0 |
|  | ≥15 | 26.3 | 24.6 | 15.2 | 25.0 | 13.1 | 24.7 |
| Ever use of oral contraceptives | No | 52.2 | 45.4 | 44.0 | 60.0 | 45.4 | 49.0 |
|  | Yes | 47.8 | 54.6 | 56.0 | 40.0 | 54.6 | 50.9 |
| Menopausal status | Pre | 22.2 | 29.5 | 40.0 | 20.0 | 27.0 | 26.1 |
|  | Peri | 7.8 | 8.8 | 10.0 | 5.0 | 3.2 | 8.2 |
|  | Post | 70.0 | 61.7 | 50.0 | 75.0 | 69.8 | 65.7 |
| HRT, current | No | 81.1 | 73.6 | 73.0 | 70.0 | 80.9 | 77.3 |
|  | Yes | 18.5 | 26.2 | 27.0 | 30.0 | 18.7 | 22.4 |
|  | Missing | 0.3 | 0.2 | 1.6 | 0.0 | 0.0 | 0.3 |
| Alcohol consumption (g/d) | 0 | 8.2 | 5.1 | 7.0 | 5.0 | 7.9 | 6.8 |
|  | <15 | 64.4 | 64.1 | 56.0 | 45.0 | 68.3 | 63.9 |
|  | 15-30 | 13.1 | 14.7 | 20.0 | 30.0 | 9.5 | 14.1 |
|  | >30 | 2.2 | 4.7 | 3.0 | 0.0 | 1.6 | 3.2 |
|  | Infrequent | 12.1 | 11.5 | 14.0 | 20.0 | 12.7 | 12.0 |
| BMI (kg‎/m²) | <20 | 5.5 | 4.4 | 4.9 | 7.0 | 3.2 | 5.0 |
|  | 20-25 | 49.0 | 46.2 | 50.9 | 47.0 | 52.4 | 47.7 |
|  | 25-30 | 34.1 | 34.9 | 32.8 | 31.0 | 30.2 | 34.4 |
|  | ≥30 | 11.5 | 14.6 | 11.3 | 15.0 | 14.3 | 12.9 |
| Time of year sample was taken | Spring | 25.6 | 28.3 | 39.0 | 25.0 | 27.0 | 27.4 |
|  | Summer | 13.5 | 17.0 | 13.0 | 20.0 | 12.7 | 15.0 |
|  | Autumn | 33.8 | 31.6 | 31.0 | 40.0 | 31.7 | 32.8 |
|  | Winter | 27.1 | 23.0 | 17.0 | 15.0 | 28.6 | 24.9 |
| Year sample was taken | 1991 | 10.0 | 8.8 | 7.0 | 10.0 | 12.7 | 9.4 |
|  | 1992 | 28.2 | 19.3 | 11.0 | 20.0 | 23.8 | 23.6 |
|  | 1993 | 30.1 | 19.4 | 22.0 | 20.0 | 17.5 | 24.8 |
|  | 1994 | 12.5 | 18.5 | 17.0 | 20.0 | 14.3 | 15.3 |
|  | 1995 | 12.3 | 21.1 | 22.0 | 15.0 | 19.0 | 16.7 |
|  | 1996 | 6.8 | 12.8 | 21.0 | 15.0 | 12.7 | 10.2 |

All data are presented as column percentage, except for mean age and mean serum zinc. Missing data <1% is not shown. Adapted from Sandsveden et al.
