# Supplementary material for: Serum zinc and dietary intake of zinc in relation to risk of different breast cancer subgroups and serum levels as a marker of intake: a prospective nested case-control study
Source: Breast Cancer Res Treat. 2021 Jul 5;189(2):571–83. doi: 10.1007/s10549-021-06318-0 (PMC8357733; doi:10.1007/s10549-021-06318-0)
Supplement: Supplementary file 4 — Supplementary file4 (DOCX 23 kb) [file 10549_2021_6318_MOESM4_ESM.docx]

Supplementary table S2. Established potential risk factors for breast cancer and quartiles of zinc intake

|  | | Dietary intake of zinc | | | | |
| --- | --- | --- | --- | --- | --- | --- |
|  |  | 1 (N=592) | 2 (N=594) | 3 (N=593) | 4 (N=593) | Total |
|  |  | 9.0 ± 0.1 ug/day | 9.5 ± 0.1 ug/day | 10.7 ± 0.1 ug/day | 19.5± 0.3 ug/day | (N=2372) |
| Age | <50 | 21.5 | 24.2 | 24.1 | 20.2 | 22.5 |
|  | 50-55 | 21.5 | 22.6 | 23.8 | 26.3 | 23.5 |
|  | 55-60 | 18.8 | 19.5 | 20.2 | 21.8 | 20.1 |
|  | ≥60 | 38.3 | 33.7 | 31.9 | 31.7 | 33.9 |
| Socio-economic index | Manual | 37.0 | 39.6 | 36.4 | 33.1 | 36.5 |
|  | Non-manual | 55.6 | 53.5 | 58.0 | 58.5 | 56.4 |
|  | Employer | 6.4 | 6.2 | 4.4 | 7.9 | 6.2 |
|  | Missing | 1.0 | 0.7 | 1.2 | 0.5 | 0.8 |
| Education | O-level college | 70.6 | 68.7 | 69.1 | 67.1 | 68.9 |
|  | A-level college | 7.1 | 8.4 | 5.9 | 7.9 | 7.3 |
|  | University | 22.0 | 22.6 | 24.8 | 25.0 | 23.6 |
| Married or cohabiting | No | 34.3 | 31.0 | 31.9 | 32.9 | 32.5 |
|  | Yes | 65.7 | 69.0 | 68.1 | 67.1 | 67.5 |
| Parity | 1 | 21.1 | 20.7 | 18.2 | 19.6 | 19.9 |
|  | 2 | 42.9 | 40.4 | 44.5 | 44.7 | 43.1 |
|  | 3 | 15.4 | 16.3 | 15.2 | 17.2 | 16.0 |
|  | 4 or more | 5.6 | 4.9 | 6.1 | 4.0 | 5.1 |
|  | Nullipara | 12.7 | 14.3 | 13.3 | 11.6 | 13.0 |
|  | Missing | 2.4 | 3.4 | 2.7 | 2.9 | 2.8 |
| Age at first childbirth | ≤20 | 16.7 | 15.5 | 18.9 | 15.2 | 16.6 |
|  | 21-25 | 35.1 | 34.8 | 31.4 | 37.4 | 34.7 |
|  | 26-30 | 24.0 | 21.7 | 25.0 | 23.6 | 23.6 |
|  | ≥31 | 9.1 | 10.3 | 8.6 | 9.3 | 9.3 |
| Age at menarche | ≤12 | 22.0 | 24.0 | 19.7 | 23.3 | 22.2 |
|  | 13-14 | 49.5 | 52.6 | 56.9 | 53.1 | 53.0 |
|  | ≥15 | 28.5 | 23.5 | 23.4 | 23.6 | 24.7 |
| Ever use of oral contraceptives | No | 55.6 | 49.8 | 45.9 | 44.9 | 49.0 |
|  | Yes | 44.4 | 50.0 | 54.1 | 55.1 | 50.9 |
| Menopausal status | Pre | 23.8 | 27.9 | 27.7 | 25.1 | 26.1 |
|  | Peri | 7.8 | 7.9 | 7.6 | 9.4 | 8.2 |
|  | Post | 68.4 | 64.1 | 64.8 | 65.4 | 65.7 |
| HRT, current | No | 75.5 | 75.3 | 81.6 | 76.7 | 77.3 |
|  | Yes | 23.5 | 24.6 | 18.4 | 23.3 | 22.4 |
| Alcohol consumption (g/d) | 0 | 7.1 | 7.2 | 7.1 | 5.7 | 6.8 |
|  | <15 | 61.7 | 63.0 | 66.3 | 64.6 | 63.9 |
|  | 15-30 | 15.2 | 14.5 | 12.8 | 13.8 | 14.1 |
|  | >30 | 5.4 | 3.0 | 2.2 | 2.4 | 3.2 |
|  | Infrequent | 10.6 | 12.1 | 11.6 | 13.5 | 12.0 |
| BMI (kg‎/m²) | <20 | 6.4 | 3.5 | 3.7 | 6.4 | 5.0 |
|  | 20-25 | 49.2 | 47.8 | 45.0 | 48.9 | 47.7 |
|  | 25-30 | 32.9 | 34.3 | 37.1 | 33.1 | 34.4 |
|  | ≥30 | 11.5 | 14.3 | 14.2 | 11.6 | 12.9 |
| Season of collection of dietary data | Jan-March | 23.8 | 22.7 | 24.5 | 28.5 | 24.9 |
|  | April-June | 26.4 | 29.5 | 25.5 | 28.0 | 27.3 |
|  | July-Sept | 17.9 | 15.2 | 16.0 | 11.1 | 15.1 |
|  | Oct-Dec | 31.9 | 32.7 | 34.1 | 32.4 | 32.8 |
| Year of collection of dietary data | 1991 | 9.5 | 9.1 | 9.4 | 9.8 | 9.4 |
|  | 1992 | 20.8 | 23.2 | 23.1 | 27.2 | 23.6 |
|  | 1993 | 24.5 | 25.4 | 25.0 | 24.5 | 24.8 |
|  | 1994 | 17.4 | 12.8 | 15.9 | 15.3 | 15.3 |
|  | 1995 | 19.4 | 17.8 | 15.3 | 14.0 | 16.7 |
|  | 1996 | 8.4 | 11.6 | 11.3 | 9.3 | 10.2 |

All data are presented as column percentage, except for mean age. Residuals are presented as the mean of total dietary intake of zinc. Missing data ≤1% is not shown.
