# Supplementary material for: Serum zinc and dietary intake of zinc in relation to risk of different breast cancer subgroups and serum levels as a marker of intake: a prospective nested case-control study
Source: Breast Cancer Res Treat. 2021 Jul 5;189(2):571–83. doi: 10.1007/s10549-021-06318-0 (PMC8357733; doi:10.1007/s10549-021-06318-0)
Supplement: Supplementary file 5 — Supplementary file5 (DOCX 14 kb) [file 10549_2021_6318_MOESM5_ESM.docx]

Supplementary table 3. Odds ratio (OR) for cases and controls in relation to serum zinc levels and dietary intake of zinc

| Group^a^ |  | Case/controls | Crude OR (CI 95) | Adjusted OR^b^ (CI 95) |
| --- | --- | --- | --- | --- |
|  |  |  |  |  |
| 1 | Low serum levels + low dietary intake | 285/248 | 1.00 | 1.00 |
|  |  |  |  |  |
| 2 | High serum levels + low dietary intake | 252/266 | 0.82 (0.65-1.05) | 0.94 (0.73-1.21) |
|  |  |  |  |  |
| 3 | Low serum levels + high dietary intake | 259/263 | 0.86 (0.67-1.09) | 0.87 (0.67-1.12) |
|  |  |  |  |  |
| 4 | High serum levels + high dietary intake | 260/277 | 0.82 (0.64-1.04) | 0.94 (0.73-1.21) |

^a^Groups of serum zinc and zinc intake with low defined as a merge of Q1 and Q2 and high defined as a merge of Q3 and Q4. Serum zinc quartiles and quartiles of zinc intake are presented in table 4.

^b^Adjusted for age, socioeconomic index, use of oral contraceptives, hormone replacement therapy, menopausal status and year of inclusion.
